# LDHC Hyperacetylation Disrupts the Guanidinoacetic Acid–Creatine Axis Underlying Cryoinjury in Boar Sperm: Rescue by GAA Supplementation

**DOI:** 10.3390/antiox15091132

**Published:** 2026-09-07

**Authors:** Shan Dou, Bo Liu, Malik Ahsan Ali, Anqi Huang, Changjun Zeng

**Affiliations:** 1 State Key Laboratory of Swine and Poultry Breeding Industry, College of Animal Science and Technology, Sichuan Agricultural University, Chengdu 611130, China; 2 Key Laboratory of Livestock and Poultry Multi-Omics, Ministry of Agriculture and Rural Affairs, College of Animal Science and Technology, Sichuan Agricultural University, Chengdu 611130, China; 3 Farm Animal Germplasm Resources and Biotech Breeding Key Laboratory of Sichuan Province, College of Animal Science and Technology, Sichuan Agricultural University, Chengdu 611130, China; 4 College of Life Science, Sichuan Agricultural University, Ya’an 625014, China; 5 Department of Theriogenology, Riphah College of Veterinary Sciences (RCVetS), Riphah International University, Lahore Campus, Lahore 54000, Pakistan

**Keywords:** boar sperm, cryopreservation, LDHC acetylation, guanidinoacetic acid, creatine biosynthesis, sperm motility, metabolomics, mitochondrial function

## Abstract

Boar sperm are highly sensitive to cryopreservation, limiting the use of frozen semen in pig breeding. We previously reported that cryopreservation alters lysine acetylation of metabolism-related proteins, including the sperm-specific isoform LDHC. Here we show that cryopreservation reduces LDH activity, and that the LDH inhibitor galloflavin recapitulates this loss, confirming that LDHC is required for boar sperm motility, acrosome integrity, and mitochondrial membrane potential. Treatment with the SIRT inhibitor nicotinamide increased LDHC acetylation and simultaneously suppressed LDH activity, indicating that hyperacetylation negatively regulates LDHC function. Untargeted metabolomics of LDH-inhibited sperm revealed a notable decrease in guanidinoacetic acid (GAA), the sole precursor of creatine. Supplementing the freezing extender with 600 μM GAA significantly improved post-thaw motility, preserved mitochondrial membrane potential and ATP levels, reduced oxidative stress, and protected acrosome integrity. These improvements were accompanied by elevated intracellular creatine content, enhanced antioxidant capacity (increased T-AOC, CAT, and SOD activities, decreased MDA), and reduced apoptosis. Our findings demonstrate that cryopreservation-induced LDHC hyperacetylation impairs enzyme activity and sperm quality, and that GAA replenishment effectively counteracts this injury through creatine-dependent and antioxidant mechanisms.

## 1. Introduction

The increasing global demand for meat and dairy products has placed new demands on animal reproductive efficiency, prompting the widespread application of assisted reproductive technologies such as artificial insemination (AI) in livestock production systems [1,2]. Although cryopreservation substantially prolongs semen storage time and facilitates cross-regional gene exchange and genetic diversity, its practical utilization rate in commercial pig farming remains less than 1% [3]. Unlike other mammalian sperm, boar sperm are characterized by a low phosphatidylcholine content and elevated levels of sphingomyelin and phosphatidylethanolamine in the plasma membrane, rendering them inherently more susceptible to temperature fluctuations and cryoinjury [4,5]. At the molecular level, cryopreservation-induced damage in porcine spermatozoa results in impaired mitochondrial activity, markedly elevated reactive oxygen species (ROS) levels, and a significant increase in the apoptosis rate, ultimately leading to reduced motility and fertilization capacity [6,7]. Compared with insemination using fresh semen, the use of frozen–thawed semen results in a 20~30% decrease in fertilization rate and a lower litter size [8,9].

In mature spermatozoa, the replacement of the majority of histones by protamines renders transcriptional and translational activities nearly silent, thereby establishing post-translational modifications (PTMs) as the predominant regulatory mechanism governing sperm protein function [10,11]. While phosphorylation has long been recognized for its role in regulating sperm capacitation [12,13], recent studies have increasingly highlighted the critical importance of acetylation in the modulation of sperm function. In mice, lysine hyperacetylation induced by deacetylase inhibitors has been shown to affect sperm motility and capacitation [14]. However, lysine acetylation induced by elevated glucose concentrations has been implicated in the regulation of sperm motility and acrosome integrity during liquid storage of boar sperm [15]. Furthermore, the acetylome of mature human sperm has revealed hundreds of lysine acetylation sites on non-histone proteins, the majority of which are localized in the mitochondria [16]. Similarly, our previous work has demonstrated that cryopreservation induces differential acetylation of numerous metabolism-related proteins in porcine spermatozoa [17]. Collectively, these findings underscore lysine acetylation as a pivotal regulatory factor in the maintenance of mature sperm function.

Sperm motility, capacitation, and the acrosome reaction all require a stable supply of ATP. Glycolysis is the primary metabolic pathway through which porcine sperm utilize glucose [18]. Maintenance of porcine sperm motility depends on ATP supplied cooperatively by glycolysis and oxidative phosphorylation (OXPHOS), with the ability to flexibly shift between metabolic pathways according to the prevailing environment [19]. ATP production in fresh sperm relies more heavily on OXPHOS; however, OXPHOS activity declines markedly while glycolysis remains relatively stable during liquid storage, suggesting that preservation stress can induce a shift in the dominant energy metabolism pathway [20,21]. Lactate dehydrogenase (LDH) is a key enzyme regulating sperm energy metabolism, and its sperm-specific isoenzyme, LDHC, accounts for over 80% of total LDH activity [22]. LDHC has been demonstrated in humans, mice, and horses to be intimately associated with sperm motility, capacitation, and fertilization competence [23,24,25,26]. Nevertheless, the role of LDHC in porcine sperm has yet to be reported.

Guanidinoacetic acid (GAA) is the direct and sole precursor for creatine synthesis in animals [27]. Previous studies have shown that dietary supplementation with GAA in roosters significantly improves sperm concentration and fertilization rates [28]. However, whether the addition of GAA to porcine semen or cryopreservation extenders can enhance sperm quality and alleviate cryoinjury remains unclear. In the present study, based on the acetylation sites we previously identified on porcine sperm LDHC, we further investigated the effect of cryopreservation-induced upregulation of LDHC acetylation on its enzymatic activity and sperm function, and explored the impact of varying concentrations of GAA supplemented to the cryopreservation extender on porcine sperm quality parameters. Our results demonstrate that acetylation of LDHC negatively regulates its enzymatic activity, leading to a decline in sperm quality parameters and a reduction in seminal GAA levels. Supplementation of the cryopreservation extender with GAA significantly improved sperm quality parameters by increasing the creatine concentration in semen. These novel findings indicate the potential application of GAA as an additive in semen cryopreservation extenders to mitigate cryoinjury.

## 2. Materials and Methods

### 2.1. Animal Ethics Statement

All animal procedures were approved by the Institutional Animal Care and Use Committee of Sichuan Agricultural University (Permit No. 20230163, approved on 14 March 2023) and were conducted in accordance with China’s Regulations for the Administration of Affairs Concerning Experimental Animals (Ministry of Science and Technology, China, 2017).

### 2.2. Semen Collection and Treatment

Ejaculates (*n* = 6) were collected from healthy, sexually mature Duroc boars aged 18–24 months using the gloved-hand method (Pengzhou Jinzhu Farm, Chengdu, China). Only ejaculates with sperm viability ≥ 90%, total motility ≥ 80%, morphologically normal spermatozoa, and sperm concentration ≥ 1 × 10^8^ sperm/mL were used. Sperm morphology was screened by light-microscopic examination, and ejaculates with obvious morphological abnormalities were excluded.

For metabolomic analysis, semen samples from the six individual boars (*n* = 6) were processed separately to preserve biological variability. For all other experiments, to minimize individual variability, ejaculates from two boars were randomly paired and pooled to generate three biological replicates (*n* = 3 pools). Each individual or pooled semen sample was centrifuged at 1200 rpm for 5 min at room temperature, and the sperm pellet was washed twice with Beltsville thawing solution (BTS) extender to remove seminal plasma. The sperm concentration was then adjusted to 1 × 10^8^ sperm/mL with BTS. For metabolomic analysis, each individual sample was divided into two equal aliquots assigned to the fresh control and galloflavin-treated groups. For the remaining experiments, each pooled sample was equally divided into aliquots for the fresh control, galloflavin treatment, nicotinamide treatment, and cryopreservation (with and without GAA supplementation) groups.

### 2.3. Analysis of Sperm Quality Parameters

#### 2.3.1. Sperm Motility Detection

A computer-assisted semen analyzer (CASA, Minitube, Tiefenbach, Germany) was used for the sperm motility detection of all samples. The sperm counting chamber (Leja^®^, Nieuw-Venep, The Netherlands) was pre-heated at 37 °C on the hot stage of the system, and the CASA was debugged until the camera image was clear [29]. Four microliters of semen were added on the pre-heated sperm counting chamber, and five random visual fields containing more than 200 sperm were selected for total and progressive motility.

#### 2.3.2. Detection of Mitochondrial Membrane Potential (ΔΨm)

ΔΨm was evaluated using JC-1 Mitochondrial Membrane Potential Detection Kit (Solarbio, Beijing, China), according to the manufacturer’s instructions. Briefly, the sperm were stained with JC-1 (5,5,6,6-tetrachloro-1,1,3,3-tetraethylbenzimidazolyl carbocyanine iodide) and incubated at 37 °C in the dark for 20 min. Then, the samples were centrifuged at 4000 rpm at 4 °C for 5 min, and the supernatant was discarded. After that, the precipitate was washed by PBS 2 times and finally resuspended in the PBS for evaluation. A flow cytometer (FACSVerse, BD Biosciences, Franklin Lakes, NJ, USA) was used to evaluate the ΔΨm of each sample.

#### 2.3.3. Detection of ROS Content

The amount of ROS in sperm cells was detected by DCFH-DA staining [30]. The experiment was performed by following the instructions provided in the manual of the Reactive Oxygen Species Assay Kit (CA1410, Solarbio, Beijing, China). Briefly, the sperm samples were centrifuged at 800× *g* for 5 min, and the supernatant was discarded. Then, the sperms were resuspended in 200 μL working solution of Dichlorodihydrofluorescein diacetate (DCFH-DA) and incubated at 37 °C in the dark for 30 min. Sperms were rinsed three times with PBS and then resuspended in the PBS for evaluation. A flow cytometer (FACSVerse, BD Biosciences, Franklin Lakes, NJ, USA) was used to evaluate the DCF fluorescence level generated from the non-fluorescent DCFH oxidation.

#### 2.3.4. Detection of ATP Content

Sperm ATP content was measured using the CellTiter-Glo^®^ Luminescent Cell Viability Assay Kit (Promega Corporation, Madison, WI, USA) according to our established method [31], with appropriate adjustments. Dilute the ATP substrate using the buffer provided in the kit, mix well to dissolve and store in portions at −20 °C; dilute the ATP standard with sterile water to 0, 0.25, 0.5, 1, and 2 μmol/L; pre-equilibrate the ATP working solution for 30 min at room temperature. Next, 20 μL of working solution was taken in a 96-well enzyme plate (opaque), 20 μL of semen was added and mixed, and a blank well was set up as a control during the experiments; the 96-well plate was incubated on a shaking table (180 r/min, 2 min) after the addition of samples. Following 10 min incubation at room temperature, the luminescence signals were measured in triplicate in 96-well plates using a multimode microplate reader (Thermo Fisher Scientific, Waltham, MA, USA). The ATP content in the semen samples was calculated from an ATP standard curve.

#### 2.3.5. Assessment of Acrosomal Status

After GF treatment for 12 h, sperm was washed and then fixed with paraformaldehyde for 10 min. Peanut agglutinin (PNA) was used to measure the acrosomal status according to the method as previously described [32]. Sperm was cultured with FITC-PNA (Sigma-Aldrich, St. Louis, MO, USA) working fluid (20 μg/mL) at 37 °C for 20 min and then 1 μL of PI (Sigma-Aldrich, USA) solution was added into the mixture and co-incubated at 37 °C for 5 min. The samples were washed three times with PBS. Epifluorescence microscopy (Olympus, Tokyo, Japan) was used to evaluate the acrosomal status of sperm with no less than 200 sperm per replicate and ImageJ was used to overlap the red and green fluorescence.

### 2.4. Detection of LDH Enzyme Activity

The LDH enzyme activity was evaluated using a Lactate Dehydrogenase (LDH) Activity Assay Kit (Solarbio, Beijing, China), according to the manufacturer’s instructions. Briefly, the semen sample was centrifuged at 4000 r/min for 5 min at room temperature and the supernatant was immediately discarded, then the precipitate was washed with PBS two times. Next, 1 mL of LDH Adsorption Solution was dropped onto the precipitate and ultrasonicate to break it up under an ice bath and then centrifuged (8000 rpm at 4 °C for 10 min) to collect the supernatant for the following measurement. New EP tubes containing different groups of lysates and different concentrations of standard or distilled water (20 μL) were set up, including sample tubes, corresponding control tubes for each sample and standard tubes containing standards. The absorbance was measured at 450 nm. Finally, the LDH enzyme activity was calculated from a standard curve.

### 2.5. Western Blot

Western blotting was performed with minor modifications as previously described [17]. Sperm proteins were extracted using RIPA lysis buffer (Beyotime Biotechnology, B0013B, Shanghai, China) and quantified with a BCA protein assay kit (Beyotime, P0010). Equal amounts of protein were resolved on 10% pre-cast Hepes SDS-PAGE gels (Beyotime, P0508S) and transferred to PVDF membranes (Beyotime, FFP32). Membranes were blocked for 1 h at room temperature with blocking buffer (Beyotime, P0252) and then incubated overnight at 4 °C with primary antibodies: anti-LDHC (1:1000; ABclonal, A15003, Wuhan, China), anti-β-tubulin (1:1000; Proteintech, 10094-1-AP, Wuhan, China), anti-GAMT monoclonal antibody (1:1000; Proteintech, 66322-1-Ig), and anti-MtCK monoclonal antibody (1:1000; Immunoway, YM1112, Suzhou, China). After three washes with TBST, membranes were incubated with HRP-conjugated goat anti-rabbit IgG (1:4000; Proteintech, SA00001-2) or HRP-conjugated goat anti-mouse IgG (1:4000; Proteintech, SA00001-1) according to the host species of the primary antibody for 1 h at 37 °C. Chemiluminescent detection was carried out using the BeyoEcl Moon kit (Beyotime, P0018FS), and signals were captured with an eBlot Touch Imager (Yibote Optoelectronic Technology, Shanghai, China). Band intensities were quantified using ImageJ software (v1.52).

### 2.6. Acetylation Detection of LDHC

The relative acetylation level of LDHC was assessed using the Signal-Seeker™ Acetyl-Lysine Detection Kit (Cytoskeleton, Denver, CO, USA) following the manufacturer’s protocol. Briefly, sperm were pelleted by centrifugation (4000 rpm, 5 min) and lysed in the BlastR Lysis Buffer provided. Protein concentration was adjusted to 1 mg/mL, and 20 μL of the lysate was saved as input. Acetylated proteins were enriched by incubating the remaining lysate with acetyl-lysine affinity beads overnight at 4 °C with rotation. The beads were washed twice with the supplied wash buffer and once with TBST, then transferred to a spin column. Bound proteins were eluted with 30 μL of bead elution buffer for 5 min and collected by centrifugation (10,000× *g*, 5 min, room temperature). LDHC acetylation was subsequently analyzed by Western blotting using anti-LDHC antibody (1:1000, ABclonal, A15003).

### 2.7. Metabolomic Profiling

Semen samples (*n* = 6) were each divided into two aliquots. One aliquot was treated with 200 μM galloflavin (GF), while the other served as the untreated control. After incubation at 17 °C for 12 h, samples were processed for metabolomic profiling. Metabolites were extracted separately for hydrophilic and hydrophobic fractions as previously described [33] and analyzed on an LC-ESI-MS/MS system consisting of a Shim-pack UFLC SHIMADZU CBM A UPLC coupled to a QTRAP 6500^+^ mass spectrometer (SCIEX, Framingham, MA, USA).

### 2.8. Semen Cryopreservation

Fresh semen collected from Duroc boars was initially diluted with Beltsville thawing solution (BTS) and centrifuged at 17 °C, 1200 rpm for 5 min. After removal of the supernatant, the sperm pellet was resuspended in an equal volume of pre-cooled Extender I (for the treatment groups, Extender was supplemented with GAA at the indicated concentrations). The sperm-extender mixture was gently mixed, placed in a beaker containing pre-cooled water at 17 °C, and transferred to a 4 °C refrigerator for slow cooling, with gentle inversion every hour. Once cooled, an equal volume of isothermal Extender II was added to the sperm suspension (final concentrations of egg yolk and glycerol were 10% and 3%, *v*/*v*, respectively). The extended semen was then loaded into 0.25 mL straws and sealed. Straws were placed horizontally on a metal rack in a liquid nitrogen vapor for 15 min before being plunged directly into liquid nitrogen for storage. Thawing was performed by immersing the straws in a 37 °C water bath for 30 s, after which the thawed semen was immediately transferred into 1.5 mL tubes containing pre-warmed BTS for subsequent analyses.

### 2.9. Determination of T-AOC, CAT, SOD Activity, and MDA Content

Total antioxidant capacity (T-AOC) was measured using a ferric reducing antioxidant power (FRAP) assay kit (Solarbio Science & Technology Co., Ltd., Beijing, China; Cat. No. BC1315). Sperm samples were homogenized in ice-cold PBS and centrifuged at 10,000× *g* for 10 min at 4 °C. The supernatant (6 μL per reaction) was incubated with pre-warmed FRAP working solution at 37 °C for 30 min in the dark, and the absorbance was recorded at 593 nm. T-AOC was calculated from a FeSO_4_ standard curve and expressed as μmol/mL.

Catalase (CAT) activity was determined using a CAT activity assay kit (Solarbio; Cat. No. BC0205). Supernatants were prepared as described above and incubated with H_2_O_2_ substrate at 25 °C. The decrease in absorbance at 240 nm was monitored, and one unit of CAT activity was defined as the amount of enzyme catalyzing the decomposition of 1 μmol H_2_O_2_ per minute. CAT activity was expressed as U/mL.

Superoxide dismutase (SOD) activity was assessed with an SOD assay kit (Solarbio; Cat. No. BC0170) based on the nitroblue tetrazolium (NBT) photochemical reduction method. Sperm supernatants were incubated with the reaction mixture containing xanthine, xanthine oxidase, and NBT at 37 °C for 30 min. Absorbance was measured at 560 nm. One unit of SOD activity was defined as the amount of enzyme required to inhibit NBT reduction by 50%, and results were expressed as U/mL.

Malondialdehyde (MDA) content, a marker of lipid peroxidation, was measured using an MDA assay kit based on the thiobarbituric acid reactive substances (TBARS) method (Solarbio; Cat. No. BC0025). Sperm samples were mixed with the thiobarbituric acid working solution and incubated at 95 °C for 60 min. After cooling and centrifugation, the absorbance of the supernatant was measured at 532 nm. MDA content was calculated from a 1,1,3,3-tetramethoxypropane standard curve and expressed as nmol/mL.

### 2.10. Evaluation of Sperm Apoptosis by Flow Cytometry

Semen (1 mL) was centrifuged at 1200 r/min for 5 min at 4 °C. The supernatant was discarded, and the sperm pellet was washed three times with PBS. Approximately 1 × 10^5^ spermatozoa were resuspended and centrifuged again under the same conditions. After removal of the supernatant, the cells were gently resuspended in 195 μL of Annexin V-FITC binding buffer (Beyotime Biotechnology, Shanghai, China; C1062L) by careful pipetting. The cell suspension was then stained with 5 μL of Annexin V-FITC and 10 μL of propidium iodide (PI) at 20~25 °C for 10~20 min in the dark. Following incubation, the samples were placed on ice and immediately subjected to flow cytometry analysis. For each sample, no fewer than 10,000 events were acquired. Viable cells (Annexin V^−^/PI^−^), early apoptotic cells (Annexin V^+^/PI^−^), late apoptotic cells (Annexin V^+^/PI^+^), and necrotic cells (Annexin V^−^/PI^+^) were discriminated.

### 2.11. Creatine Content Assay

Creatine content was measured using a commercial creatine assay kit (Solarbio Science & Technology Co., Ltd., Beijing, China; Cat. No. BC4920) based on the enzymatic conversion of creatine to sarcosine and urea with subsequent colorimetric detection, following the manufacturer’s instructions. Sperm samples were homogenized in ice-cold extraction buffer and centrifuged at 10,000× *g* for 10 min at 4 °C. The supernatant was incubated with the reaction working solution at room temperature for 10 min in the dark. Absorbance was measured at 530 nm using a microplate reader. Creatine content was calculated from a creatine standard curve and expressed as μg per mL of sperm sample (μg/mL).

### 2.12. Measurement of NAD^+^ and NADH

Intracellular levels of NAD^+^ and NADH were determined using the NAD^+^/NADH assay kit (Beyotime Biotechnology, Shanghai, China; S0175), which employs a WST-8-based enzymatic cycling method for colorimetric quantification. Sperm samples were collected, washed in ice-cold PBS (pH 7.4), and lysed on ice with the extraction buffer provided. After centrifugation (12,000× *g*, 10 min, 4 °C), the supernatant was harvested and split into two portions. To achieve selective detection of NADH, one portion was heated at 60 °C for 30 min to thermally decompose NAD^+^, whereas the other was kept on ice to preserve total NAD^+^ plus NADH. Both portions were then mixed with the working solution containing WST-8, alcohol dehydrogenase, and electron coupling reagents, followed by incubation at 37 °C for 30 min in the dark. The absorbance was measured at 450 nm using a microplate reader. A standard curve was generated with NADH standards (0–10 μM) run in parallel. NAD^+^ levels were obtained by subtracting NADH values from the total NAD^+^ + NADH readout. Results are expressed as micromolar (μM) concentrations in the cell extracts.

### 2.13. Statistical Analysis

All experiments were conducted independently with at least three independent biological replicates, and data are presented as the mean ± standard error of the mean (SEM). Statistical analyses were performed using GraphPad Prism v9.0.0 (GraphPad Software, San Diego, CA, USA). Comparisons between two groups were analyzed using an independent samples *t*-test. For comparisons among three or more groups, one-way analysis of variance (ANOVA) followed by Tukey’s post hoc multiple comparisons test was employed. A value of *p* < 0.05 was considered statistically significant.

## 3. Results

### 3.1. Cryopreservation Reduces Sperm Motility Through Decreased LDHC Enzyme Activity

**Figure 1:**
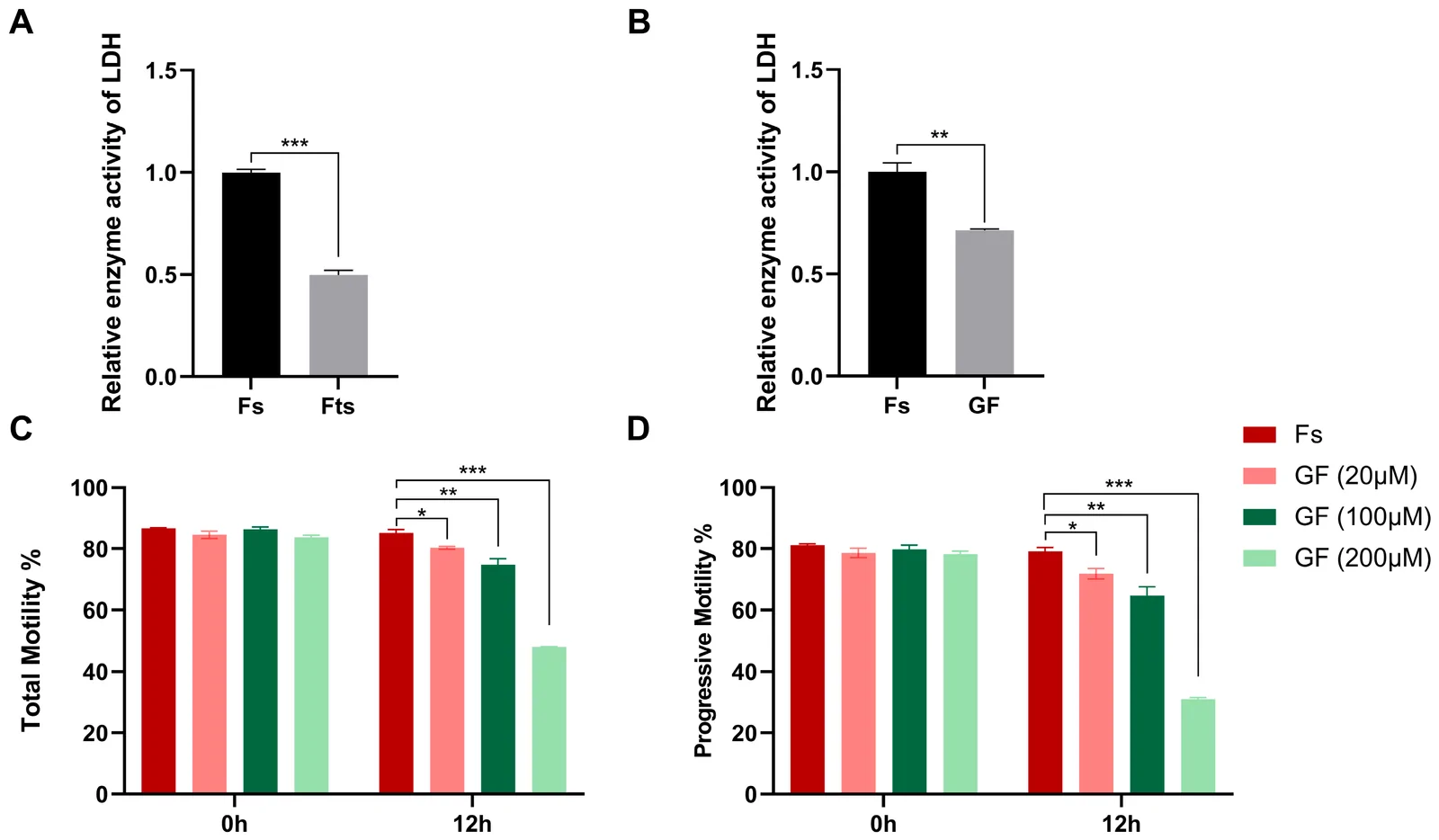
Addition of different doses of GF on LDH activity and sperm motility. (**A**) Relative LDH enzyme activity between fresh (Fs) and frozen–thawed (Fts) groups. (**B**) Relative LDH enzyme activity between fresh (Fs) and 600 μM galloflavin (GF) groups. (**C**) Total motility of sperm after different doses of GF treatment for 0 h and 12 h. (**D**) Progressive motility of sperm after different doses of GF treatment for 0 h and 12 h. Data are presented as mean ± SEM (*n* = 3). * *p* < 0.05, ** *p* < 0.01, *** *p* < 0.001.

We first observed a marked decrease (*p* < 0.01) in LDH activity in frozen–thawed sperm compared with the fresh control group (Figure 1A). To mimic the loss of LDH activity during cryopreservation, we treated sperm with varying doses of Galloflavin (GF), a selective LDH inhibitor (Figure 1B). Sperm kinetic parameters were systematically evaluated using computer-assisted sperm analysis (CASA). A dose-dependent inhibition of both total and progressive motility was observed within 12 h of exposure (Figure 1C,D), indicating that LDH activity is crucial for the maintenance of sperm motility.

### 3.2. Inhibition of LDH Activity Impairs Acrosome Integrity, Mitochondrial Membrane Potential (MMP), ROS Accumulation, and ATP Levels in Boar Sperm

**Figure 2:**
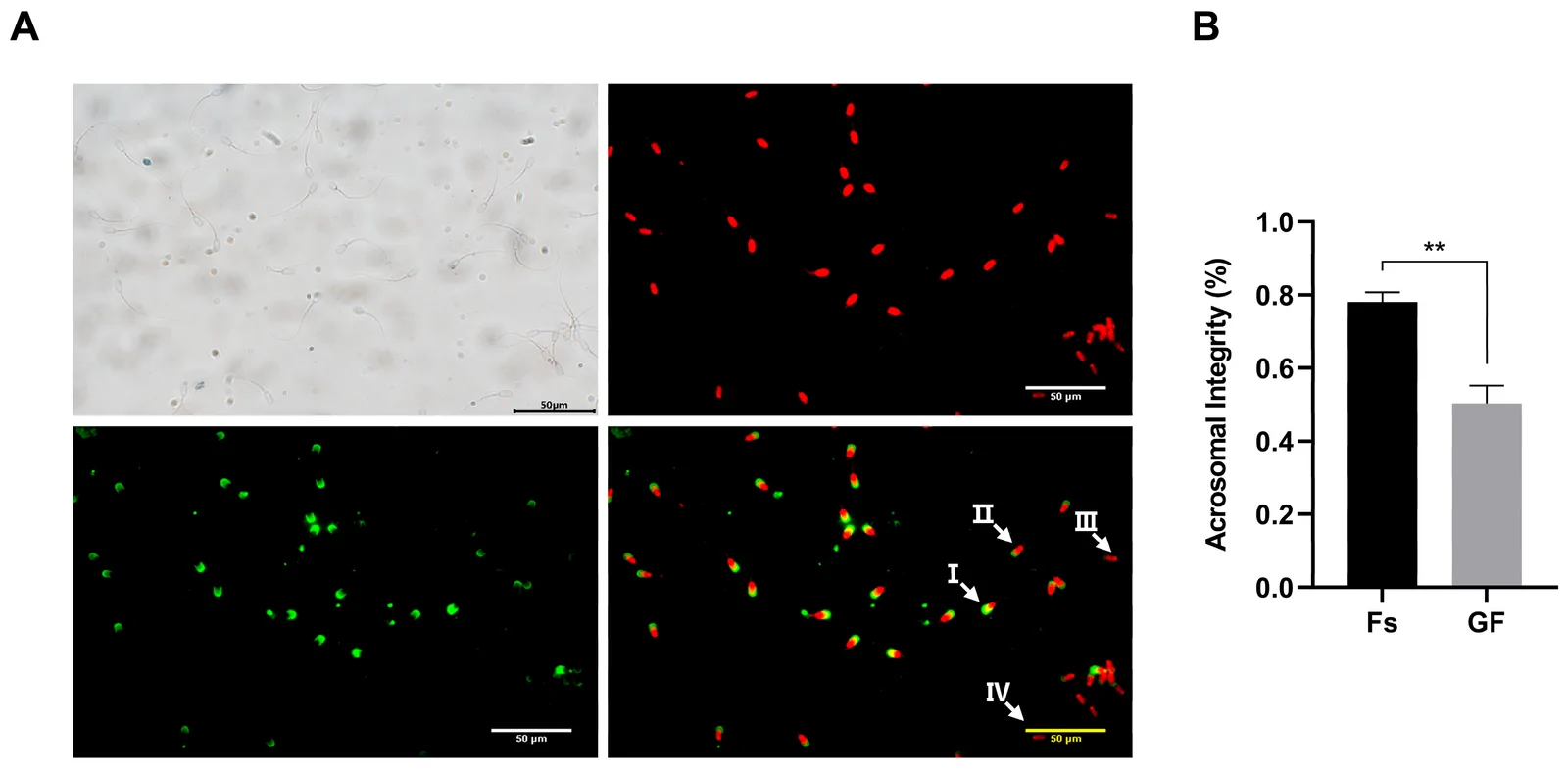
Effects of addition of GF on acrosome integrity. (**A**) Assessment of the acrosomal status and intact acrosome rate at 37 °C by a PNA/PI staining assay. I: integral acrosome (PI^+^/PNA^+^), II: acrosomal reaction (PI^+^/PNA^+^), III: damaged acrosome and incomplete membrane (PI^+^/PNA^+^). IV: dead sperm (PI^+^/PNA^−^). (**B**) Acrosomal integrity of Fs(fresh) and GF (200 μM GF) group. Data are presented as mean ± SEM (*n* = 3). ** *p* < 0.01.

**Figure 3:**
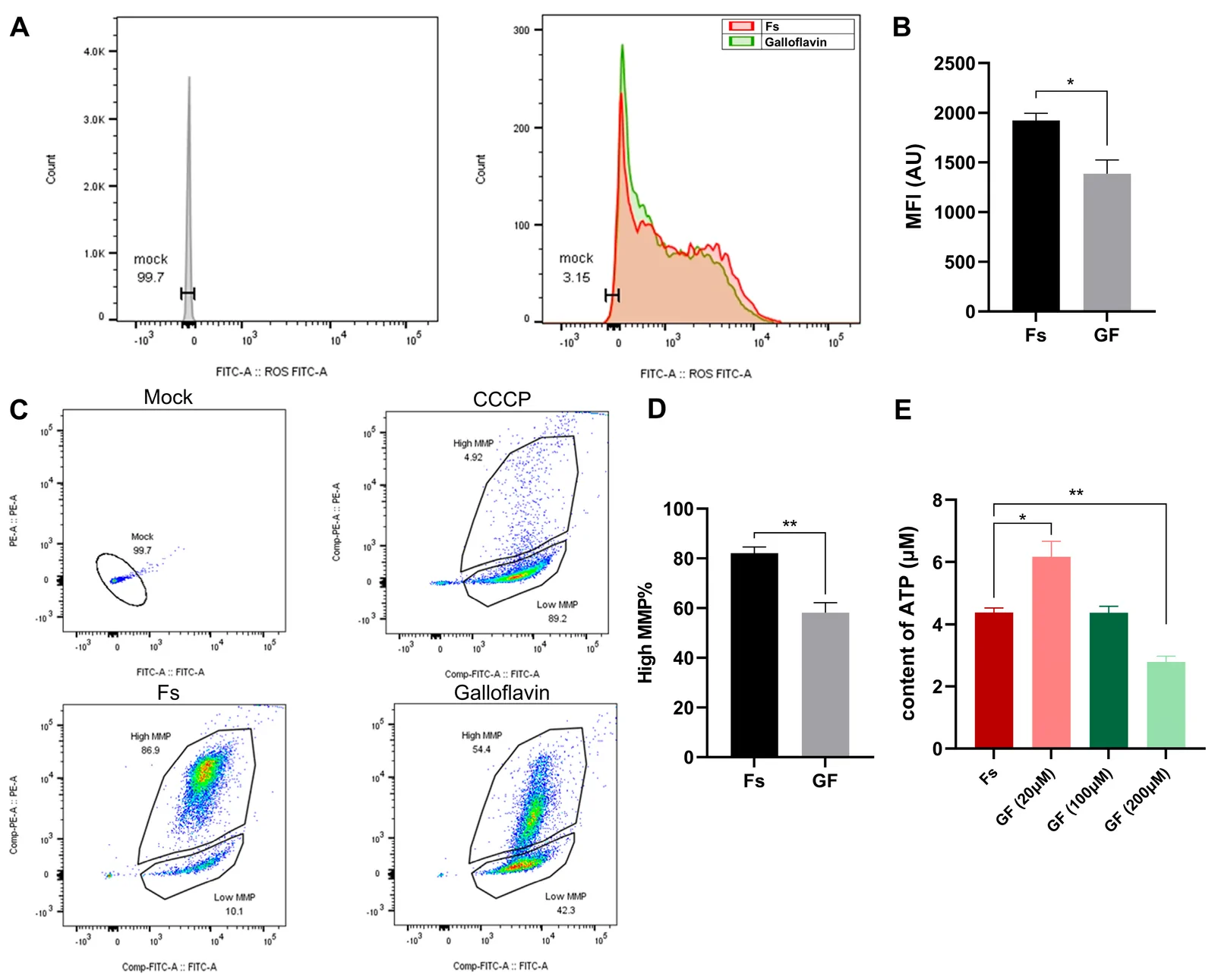
Effects of addition of GF on sperm quality parameters. (**A**) Peak plot of ROS level analyzed by flow cytometry. Mock: control group without DCFH-DA dye; the red peak shows the ROS level of the fresh group, while the green peak shows the ROS level of the 200 μM GF group. (**B**) The mean fluorescence intensity (MFI) of sperm. (**C**) Analysis of MMP by flow cytometry: upper gate shows cells with high MMP, lower gate shows cells with low MMP. Mock (control group without JC-1 dye), CCCP (positive control), Fs (fresh group), Galloflavin (200 μM GF group). (**D**) High MMP rate of sperm. (**E**) ATP concentration of sperm. Data are presented as mean ± SEM (*n* = 3). * *p* < 0.05, ** *p* < 0.01.

To further explore the consequences of LDH activity loss on sperm fertilization capacity and overall quality, we assessed acrosome integrity, mitochondrial membrane potential (MMP), reactive oxygen species (ROS) levels, and ATP levels following GF treatment. A significant decline in the percentage of acrosome-intact sperm was observed in the 200 μM GF group at 12 h compared to the fresh control (Figure 2), indicating a reduced fertilization capacity. ROS, which are oxygen-containing molecules generated during cellular metabolism, play a dual role in sperm physiology: physiological levels are critical for metabolism and signaling, whereas excessive ROS production causes damage to the sperm plasma membrane. Our results revealed a significant increase (*p* < 0.05) in ROS levels in the GF-treated group relative to the fresh control at 12 h (Figure 3A,B). Moreover, a significant decrease in MMP was detected after 12 h of GF treatment (Figure 3C,D). With respect to ATP content, which directly reflects mitochondrial activity, low GF concentrations significantly increased ATP levels (*p* < 0.05), whereas high concentrations caused an extreme decrease (*p* < 0.01) compared with the fresh group (Figure 3E). Collectively, these results suggest that pharmacological LDH inhibition compromises sperm fertilization capacity and metabolic activity. However, although galloflavin is widely used as a selective LDH inhibitor, we cannot exclude the possibility that some of these changes reflect off-target effects. These data therefore support, rather than definitively prove, a specific LDHC-mediated mechanism, and this interpretation is reinforced by the acetylation-related evidence presented in the following section.

### 3.3. Cryopreservation-Mediated Hyperacetylation of LDHC Impairs Sperm Quality by Decreasing Enzyme Activity

**Figure 4:**
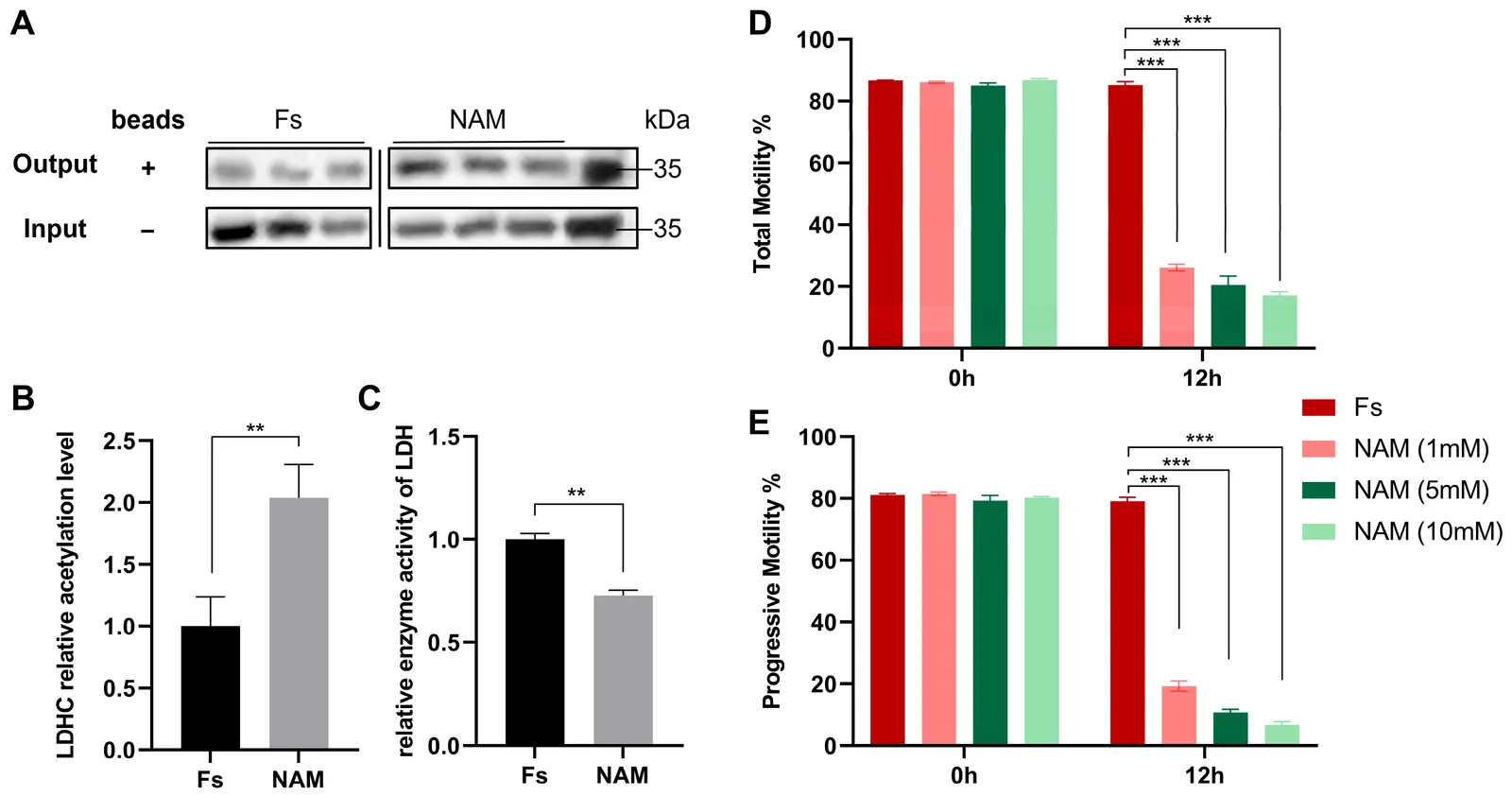
Effects of NAM treatment on LDHC acetylation level, LDH enzyme activity and sperm motility. (**A**) Western blotting identified LDHC levels between fresh sperm and sperm treated with 10 mM NAM for 12 h with (Output/+) or without (Input/−) the acetyl-lysine affinity beads. (**B**) Relative acetylation level (Output/Input) of LDHC between fresh sperm and sperm treated with 10 mM NAM for 12 h. (**C**) The relative enzyme activity of LDH between fresh sperm and sperm treated with 10 mM NAM for 12 h. (**D**) Total motility of sperm after different doses of NAM treatment for 12 h. (**E**) Progressive motility of sperm after different doses of NAM treatment for 12 h. Data are presented as mean ± SEM (*n* = 3). ** *p* < 0.01, *** *p* < 0.001.

**Figure 5:**
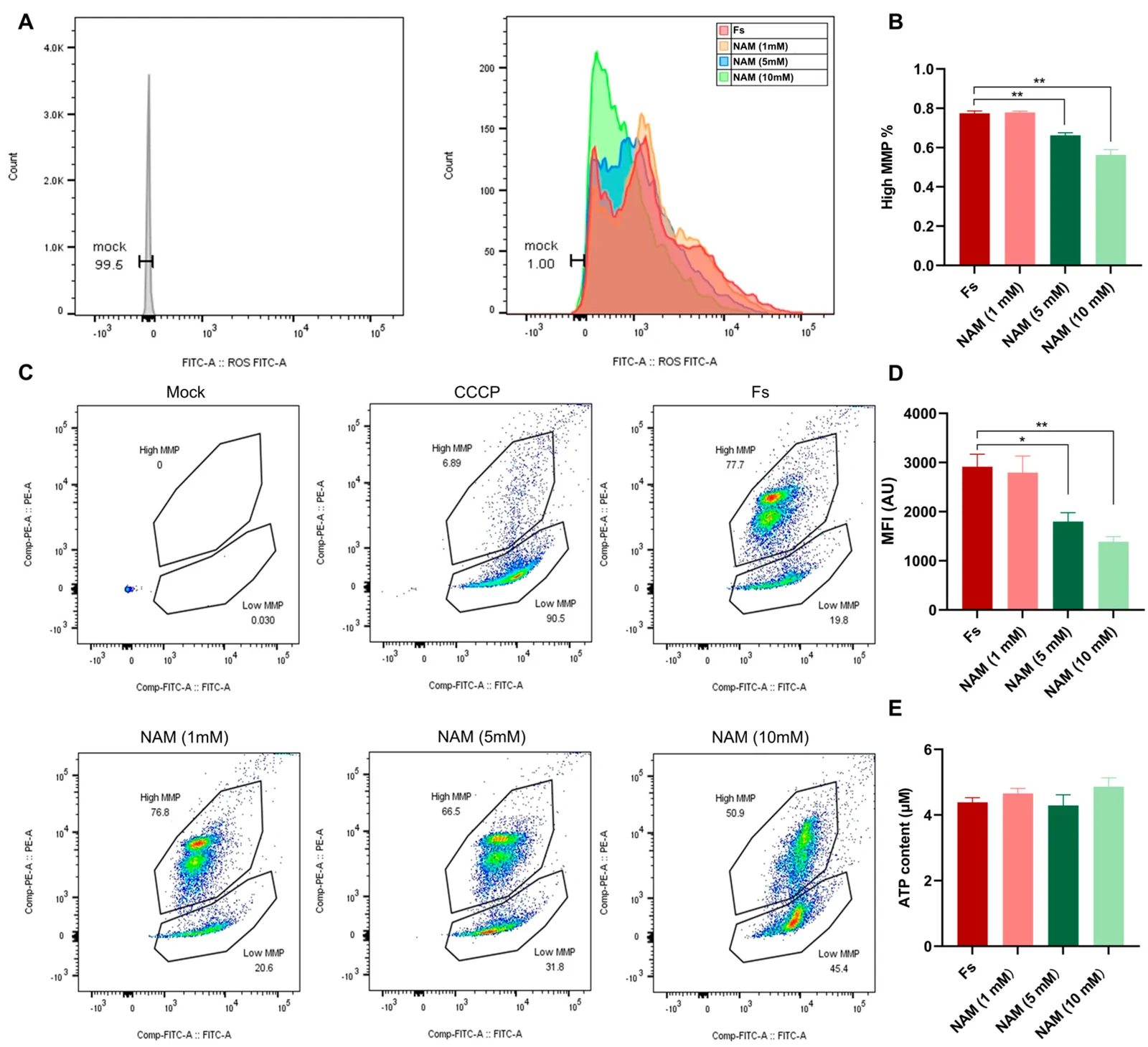
Effects of NAM addition on boar sperm quality. (**A**) Peak plot of ROS levels analyzed by flow cytometry. Mock: control group without DCFH-DA dye; the red peak shows the ROS level of the fresh group, while the other peaks show the ROS levels of sperm treated with different concentrations of NAM for 12 h. (**B**) The mean fluorescence intensity (MFI) of sperm. (**C**) Analysis of MMP by flow cytometry: upper gate shows cells with high MMP, lower gate shows cells with low MMP. Mock: control group without JC-1 dye; CCCP (positive control); Fs (fresh group). (**D**) High MMP rate of sperm. (**E**) ATP concentration of sperm. Data are presented as mean ± SEM (n = 3). * *p* < 0.05, ** *p* < 0.01.

Our previous study showed that LDHC is among the top 10 differentially acetylated proteins (DAPs) involved in regulating energy metabolism in boar sperm when comparing fresh and frozen–thawed samples [17]. In pancreatic cancer cells, the acetylation level of LDHA negatively regulates its enzymatic activity and is controlled by the SIRT2 deacetylase [34]. To investigate the regulatory mechanism of LDHC acetylation in boar sperm and whether elevated acetylation affects its enzyme activity, we incubated semen with 10 mM nicotinamide (NAM), a selective SIRT deacetylase inhibitor, for 12 h. The relative acetylation level was then assessed using the Signal-Seeker™ Acetyl-Lysine Detection Kit. Our results demonstrated that 10 mM NAM treatment significantly up-regulated LDHC acetylation (Figure 4A,B), and LDH enzyme activity was negatively correlated with LDHC acetylation levels (Figure 4C). Having established that LDH activity is linked to sperm quality and fertilizing ability, we next examined the effect of a NAM concentration gradient on sperm parameters. A significant dose-dependent reduction in both total and progressive motility was observed (Figure 5D,E). Similarly, ROS levels were significantly lower than in the fresh group after 5 mM or 10 mM NAM treatment for 12 h (Figure 5A,B), and a comparable trend was observed for MMP (Figure 5C,D). No significant differences (*p* > 0.05) were detected in sperm ATP content between any of the NAM-treated groups and the fresh control (Figure 5E). These findings suggest that cryopreservation-mediated hyperacetylation of LDHC impairs its enzymatic activity, ultimately affecting sperm motility and quality parameters.

### 3.4. Global Metabolite Profiles Reveal Metabolic Disruption in Fresh vs. Galloflavin-Treated Sperm

**Figure 6:**
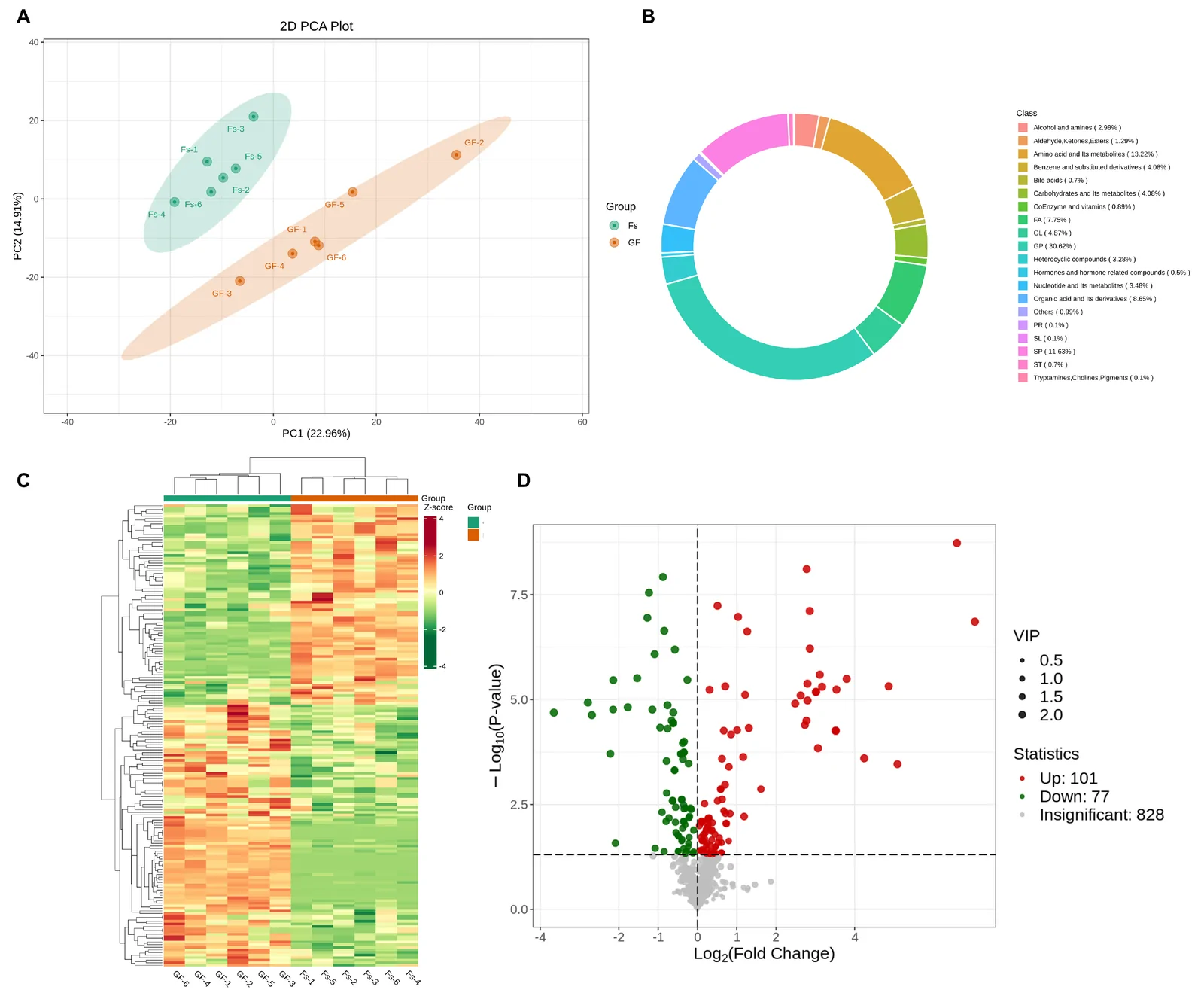
Global metabolite profiles between fresh (Fs) and galloflavin (GF) groups. (**A**) The principal component analysis of the fresh and galloflavin groups, where PC1 indicates the first principal component and PC2 indicates the second principal component. (**B**) Ring diagram of metabolite class composition; each color represents a metabolite class and the area of the color block indicates the proportion of that class. (**C**) Heatmap of differential metabolites between fresh (Fs) and galloflavin (GF) groups. The *x*-axis represents sample name, *y*-axis represents the normalized value of FPKM of differential metabolites, red and green represent increased and decreased expression, respectively, and the color depth represents the size of the difference in metabolites expression. (**D**) The volcano plot of all identified metabolites between fresh and galloflavin groups, where the *x*-axis represents Log_2_ (fold change), *y*-axis represents −Log_10_ (*p* value), and the size of each dot indicates the VIP value of each metabolite. Red dots: Significantly up (*p* < 0.05, Log_2_ (fold change) > 0) metabolites; Green dots: Significantly down (*p* < 0.05, Log_2_ (fold change) < 0) metabolites.

To better understand the metabolic consequences of LDH activity reduction induced by acetylation, we employed an untargeted metabolomics approach to compare metabolite profiles between groups. Principal component analysis (PCA), performed using the prcomp function in R (www.r-project.org), revealed a clear separation of the metabolome between fresh (Fs) and galloflavin (GF) groups (Figure 6A). Among the 1006 detected metabolites, the major categories included glycoproteins (30.62%), amino acids and their derivatives (13.22%), sphingolipids (SP, 11.63%), organic acids and their derivatives (8.85%), and fatty acids (FA, 7.75%); the overall distribution is shown in a ring diagram (Figure 6B). A total of 101 up-regulated and 77 down-regulated metabolites were identified, and their distribution is illustrated by a heatmap and a volcano plot (Figure 6C,D).

To further identify the key differential metabolites driven by the loss of LDH activity, we applied stringent screening criteria of q < 0.05, VIP > 1, and |log_2_FC| > 1 to select the top 20 most significantly up-regulated and down-regulated metabolites. The detailed information of these metabolites is summarized in Table 1. Among the up-regulated metabolites, dihydrocaffeic acid and ascorbic acid exhibited the most pronounced fold changes, with q-values approaching zero, indicating exceptionally high statistical confidence. Other notably up-regulated metabolites included thioguanine, nicotinamide-N-oxide (log_2_FC = 4.87), and glycocholic acid (log_2_FC = 4.24). In the down-regulated category, imidazole-4-methanol (log_2_FC = −3.65), LPS (20:4/0:0) (log_2_FC = −2.79), and O-acetyl-L-homoserine (log_2_FC = −2.69) represented the most sharply decreased metabolites. Notably, guanidineacetic acid (GAA; log_2_FC = −1.23, VIP = 2.21, q = 7.17 × 10^−6^), the sole precursor for creatine biosynthesis, was significantly down-regulated, suggesting that LDH activity loss disrupts the GAA–creatine metabolic axis in boar sperm. Collectively, these differentially expressed metabolites reflect a profound metabolic reprogramming triggered by diminished LDH activity.

### 3.5. GAA Supplementation Alleviates Cryoinjury and Improves Sperm Quality Parameters

**Figure 7:**
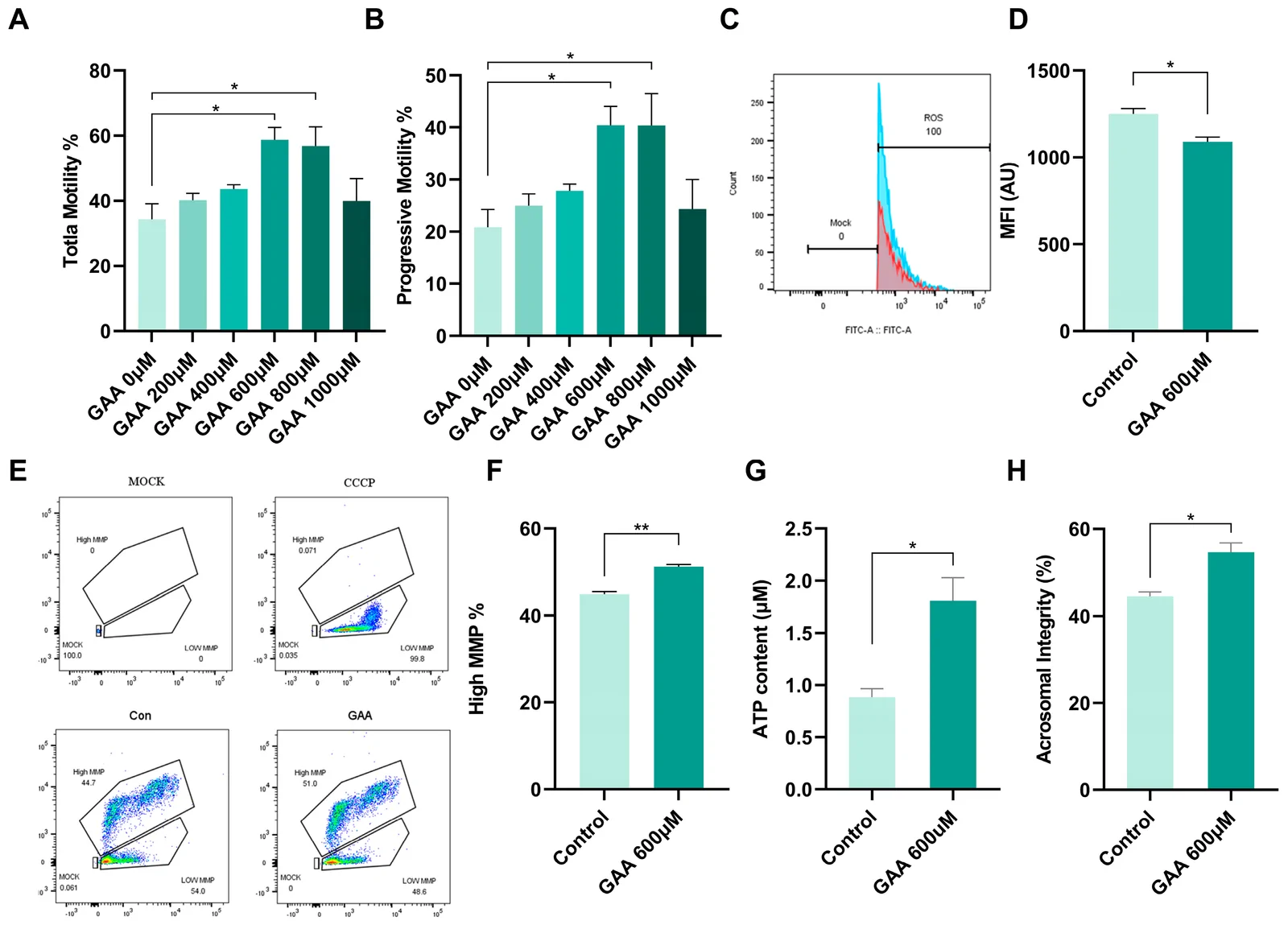
GAA supplementation alleviates cryoinjury and improves the quality parameters of frozen–thawed boar sperm. (**A**) Total motility of frozen–thawed sperm treated with varying concentrations of GAA. (**B**) Progressive motility under the corresponding GAA concentration gradient. (**C**) Representative flow cytometric histograms of intracellular ROS levels; the Mock group served as the negative control, the red peak indicates the 600 μM GAA treated group, and the blue peak indicates the control group. (**D**) Quantification of ROS levels expressed as mean fluorescence intensity (MFI). (**E**) Representative flow cytometric scatter plots of MMP; CCCP was used as the depolarized positive control. (**F**) Statistical analysis of the proportion of high-MMP sperm. (**G**) Intracellular ATP content. (**H**) Sperm acrosome integrity rate. Data are presented as mean ± SEM (*n* = 3). * *p* < 0.05, ** *p* < 0.01.

Given that GAA was identified as a significantly down-regulated metabolite with the lowest q-value following LDH activity inhibition, and especially considering its role as the sole precursor for creatine biosynthesis, we further evaluated whether supplementation of the cryopreservation extender with GAA could mitigate cryoinjury in boar sperm. CASA revealed that GAA exerted a dose-dependent protective effect on both total and progressive motility. Compared with the control group, motility was significantly enhanced at concentrations ranging from 600 to 800 μM (*p* < 0.05), but this beneficial effect diminished at 1000 μM, indicating a narrow therapeutic window (Figure 7A,B). Consistent with this trend, GAA treatment at 600 μM significantly reduced intracellular ROS levels compared with the control group (*p* < 0.05, Figure 7C,D), indicating an attenuation of frozen–thaw-induced oxidative stress. Moreover, the MMP level was significantly preserved in the presence of 600 μM GAA (*p* < 0.01, Figure 7E,F), accompanied by a corresponding elevation in intracellular ATP content (*p* < 0.05, Figure 7G). Furthermore, the acrosome integrity rate was significantly higher in the GAA-treated group than in the control group (*p* < 0.05, Figure 7H), suggesting that GAA protects against premature acrosomal membrane damage during cryopreservation. Collectively, these results demonstrate that GAA supplementation effectively alleviates cryoinjury, preserves mitochondrial function and energy metabolism, reduces oxidative stress, and may preserve sperm functional competence in frozen–thawed boar sperm.

### 3.6. GAA Supplementation Promotes Creatine Biosynthesis, Enhances Antioxidant Capacity, and Attenuates Apoptosis in Frozen–Thawed Sperm

**Figure 8:**
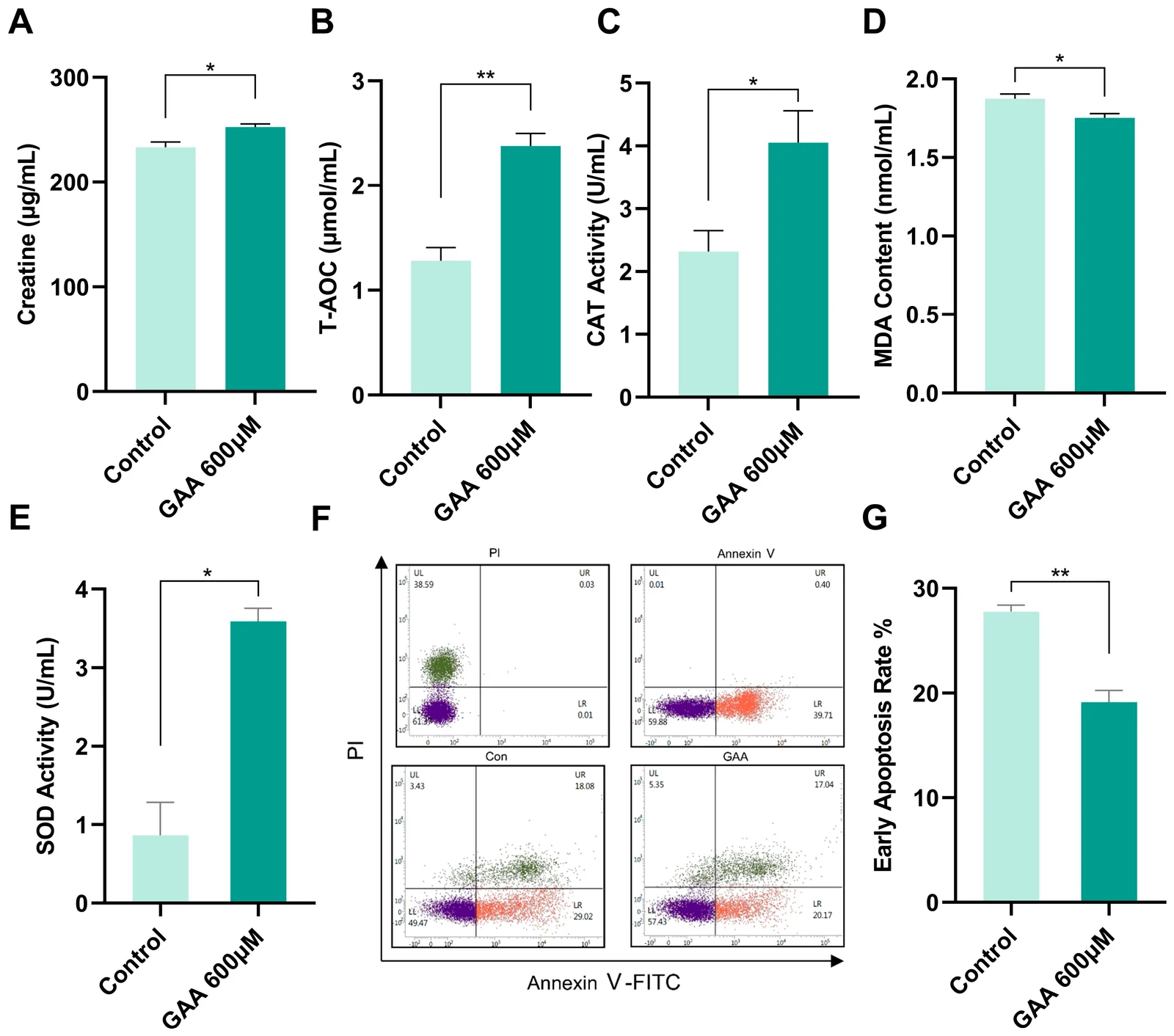
GAA supplementation promotes creatine biosynthesis, enhances antioxidant capacity, and attenuates apoptosis in frozen–thawed boar sperm. (**A**) Total creatine content in control and 600 μM GAA-treated sperm. (**B**) Total antioxidant capacity (T-AOC). (**C**) Catalase (CAT) activity. (**D**) Malondialdehyde (MDA) content. (**E**) Superoxide dismutase (SOD) activity. (**F**) Representative flow cytometric dot plots of Annexin V-FITC/PI double staining showing PI single-staining, Annexin V single-staining, untreated control, and 600 μM GAA-treated groups. (**G**) Quantification of the early apoptosis rate. Data are presented as mean ± SEM (*n* = 3). * *p* < 0.05, ** *p* < 0.01.

To investigate the protective mechanism of GAA, we first examined whether boar sperm express the enzymatic machinery required for creatine biosynthesis. Western blot analysis detected both guanidinoacetate N-methyltransferase (GAMT) and mitochondrial creatine kinase (MtCK) in fresh boar sperm (Figure S1), indicating that boar sperm possess the biochemical basis for converting GAA to creatine. Consistent with this, 600 μM GAA treatment significantly increased creatine levels compared with untreated controls (*p* < 0.05; Figure 8A), supporting the view that exogenous GAA can serve as a substrate for creatine synthesis. We next examined whether this metabolic effect was accompanied by improved antioxidant defenses. GAA-treated sperm exhibited significantly elevated total antioxidant capacity (T-AOC; *p* < 0.01), catalase (CAT) activity (*p* < 0.05), and superoxide dismutase (SOD) activity (*p* < 0.01), along with a significant reduction in malondialdehyde (MDA) content (*p* < 0.05; Figure 8B–E), demonstrating that GAA reinforces the endogenous antioxidant system and alleviates lipid peroxidation. We then assessed whether these cytoprotective changes translated into reduced apoptosis using Annexin V-FITC/PI double staining and flow cytometry (Figure 8F). Quantitative analysis confirmed a significantly lower early apoptosis rate in the GAA-treated group relative to controls (*p* < 0.01; Figure 8G). Together, these results indicate that GAA supplementation enhances creatine biosynthesis, strengthens antioxidant defenses, and suppresses cryopreservation-induced apoptosis in boar sperm.

### 3.7. GAA Supplementation Preserves the NAD^+^/NADH Redox Balance in Frozen–Thawed Sperm

**Figure 9:**
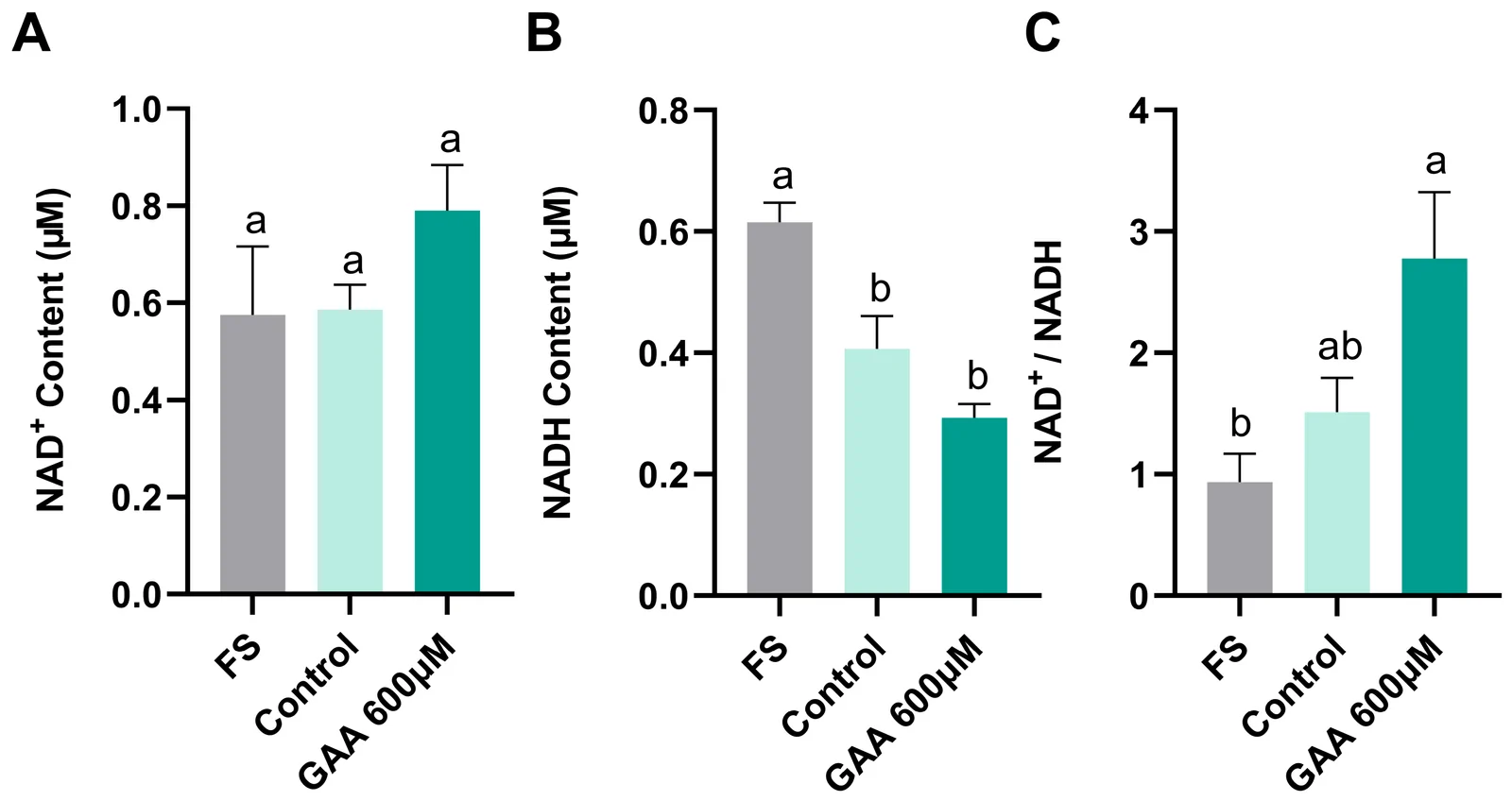
Effects of GAA supplementation on the NAD^+^/NADH redox balance in frozen–thawed boar sperm. (**A**) NAD^+^ content in fresh, frozen–thawed control, and GAA groups. (**B**) NADH content across the three groups. (**C**) NAD^+^/NADH ratio. Values are presented as mean ± SEM (*n* = 3). Different lowercase letters (a, b) indicate statistically significant differences between groups (*p* < 0.05).

To investigate whether GAA-mediated mitochondrial enhancement was linked to altered redox status, we measured NAD^+^ and NADH levels. While NAD^+^ content remained unchanged across all groups (Figure 9A), cryopreservation significantly elevated NADH levels regardless of GAA supplementation (*p* < 0.05 vs. fresh; Figure 9B), suggesting a freeze–thaw-induced shift toward a more reduced state. Although the NAD^+^/NADH ratio in the GAA-treated group was higher than that in fresh sperm (*p* < 0.05), it did not differ significantly from the frozen–thawed control group (Figure 9C). Thus, under our experimental conditions, GAA supplementation did not significantly reverse the cryopreservation-induced NADH accumulation but maintained the NAD^+^/NADH ratio at a level comparable to that of untreated frozen–thawed controls. This redox profile, while not further improved by GAA, is consistent with the preserved mitochondrial function and ATP availability observed in the GAA group.

## 4. Discussion

The present study identifies cryopreservation-induced hyperacetylation of LDHC as a cause of impaired energy metabolism in boar sperm and evaluates GAA as a cryoprotective additive capable of counteracting this injury by promoting creatine synthesis and strengthening antioxidant defenses.

LDH maintains glycolytic flux and ATP supply in sperm by catalyzing the interconversion of pyruvate and lactate. Among its isoforms, LDHC is the predominant form in mammalian sperm, accounting for more than 80% of total LDH activity [22]. Work in mice established that LDHC is essential for fertility: LDHC knockout males produce sperm that fail to swim or fertilize, yet transgenic expression of human LDHA fully rescues these defects, confirming that the catalytic function of LDHC, rather than any structural role, is what matters [23,24]. Similar functional links between LDHC and sperm motility have been reported in humans [16], stallions [25], and mice [23,24,26]. For porcine sperm, however, the literature is sparse. Apart from an early description of LDH isoenzyme shifts during epididymal maturation published over thirty years ago, the role of LDHC has not been examined. We found that galloflavin treatment caused a dose-dependent loss of total and progressive motility, reduced acrosome integrity, lowered mitochondrial membrane potential, and disrupted ATP homeostasis. These results argue that LDHC-dependent LDH activity is as important for boar sperm function as it is in other mammals.

Lysine acetylation has received considerable attention as a regulatory mechanism in mature sperm, where transcriptional activity is largely shut down [10,11]. Our previous acetylome study ranked LDHC among the top ten differentially acetylated proteins when comparing fresh and frozen–thawed boar sperm [17], which motivated the present work. In somatic cells, acetylation has been shown to suppress LDH activity: SIRT2 removes an acetyl group from LDHA at lysine-5 in pancreatic cancer cells [34], and SIRT5 deacetylates LDHB in colorectal cancer, with functional consequences for autophagy and tumor growth [35]. More directly relevant to our findings, a recent report showed that NAM administration reduces LDHC acetylation and improves spermatogenesis in obese mice, indicating that the acetylation state of LDHC carries functional significance [36]. In boar sperm, we observed that NAM treatment raised LDHC acetylation and concurrently lowered LDH activity, consistent with a negative regulatory relationship. These data extend the acetylation–LDH regulatory axis, previously described in cancer cells, to the male gamete. Nevertheless, the pathway linking cryopreservation-induced LDHC hyperacetylation to the GAA–creatine axis remains largely inferential: the GAA–creatine connection was inferred from metabolite profiling after galloflavin-induced LDH inhibition, rather than from direct manipulation of LDHC acetylation. The present data therefore support a plausible sequence of events rather than a fully established causal chain.

Our metabolomic data revealed broad shifts in metabolite profiles when LDH activity was blocked. One feature that stood out was the marked drop in GAA levels. GAA is the sole precursor for creatine biosynthesis [27], and the creatine–phosphocreatine system provides a spatial energy buffer that shuttles high-energy phosphate from mitochondria to the flagellum. This pathway has not been studied extensively in the context of sperm freezing. Dietary GAA has been reported to improve sperm output and fertility in roosters [28], but whether mammalian sperm can take up and utilize exogenous GAA under cryopreservation conditions was unknown. The depletion of GAA following LDH inhibition pointed to the GAA–creatine axis as a weak link that might be reinforced by external supplementation.

Adding GAA to the freezing extender improved post-thaw motility in a dose-dependent manner, with the best results seen between 600 and 800 μM. We confirmed that the added GAA entered the creatine synthesis pathway: total creatine levels rose in GAA-treated sperm. The improvements were not limited to motility; MMP, ATP levels, and acrosome integrity were all significantly better in the GAA group. These functional gains were accompanied by higher T AOC, CAT, and SOD activities and lower MDA levels, pointing to a broader reinforcement of antioxidant capacity. The reduction in apoptosis rate in GAA-treated sperm fits with the idea that oxidative damage is a major trigger of cell death after freezing and thawing. Several additives have been tested for boar sperm cryopreservation, including α tocopherol [37], pyrroloquinoline quinone [38], mangiferin [39], polyamines [30], and a spermidine–phosphocreatine combination [40], most of which act through antioxidant mechanisms or by supplying ATP/creatine directly. Our group previously found that L methionine also improves cryosurvival and antioxidant activity in boar sperm [33]. Alongside the current GAA data, these findings suggest that amino acid derivatives are worth further investigation as a class of cryoprotectants. Conceptually, supplying the biosynthetic precursor GAA rather than phosphocreatine itself has the appeal of relying on endogenous enzymatic conversion, which could sustain creatine production over time. That said, we did not run head-to-head comparisons of GAA against phosphocreatine, so any claim of superiority would be premature.

One unexpected observation was that cryopreservation raised NADH levels regardless of GAA treatment, pushing the redox balance toward a more reduced state. This NADH buildup may reflect impaired Complex I activity or a general decline in NADH oxidation capacity resulting from mitochondrial damage. However, Complex I activity was not directly measured in the present study, this explanation should be regarded as a hypothesis rather than an established mechanism. GAA did not reverse this shift, yet mitochondrial function and sperm motility were preserved. This apparent disconnect raises the possibility that GAA-derived creatine supports ATP distribution through the phosphocreatine shuttle without correcting the proximal redox defect. Alternatively, whole-cell NAD^+^/NADH measurements may mask distinct changes in cytosolic versus mitochondrial compartments. These observations suggest that further improvements in cryopreservation protocols might be achieved by combining GAA with agents that promote NAD^+^ regeneration or limit NADH accumulation.

Although these findings are promising, they should be interpreted with caution because the study was conducted with a limited number of animals and samples. Larger trials will be needed to confirm the generalizability of these results. Several mechanistic questions also remain open. In particular, the acetyltransferase and deacetylase pair responsible for LDHC acetylation and deacetylation has not been identified in sperm, and the functional acetylated lysine residues that control porcine sperm LDHC activity remain unknown. This issue is not merely technical. Recent work has shown that lysine acylation at specific sites can directly regulate LDH activity [41], and emerging evidence indicates that non-enzymatic acetylation also contributes to sperm protein acetylation under conditions of intracellular alkalinization [42]. These observations underscore the need to determine whether the LDHC hyperacetylation associated with cryopreservation is enzyme-driven or partly non-enzymatic and which modification sites are responsible for the loss of enzyme activity. Beyond addressing these mechanistic questions, future studies should evaluate GAA in field insemination trials, test its compatibility with established antioxidant-based extenders, and explore whether the GAA–creatine axis can be targeted in other livestock species or in human sperm cryopreservation.

In summary, we demonstrated that cryopreservation induces hyperacetylation of LDHC, leading to loss of its enzymatic activity and disruption of boar sperm energy metabolism. We identified GAA as a metabolite depleted under these conditions and demonstrated that its replenishment in the freezing extender reduces cryoinjury through creatine dependent and antioxidant mechanisms.

## Figures and Tables

**Table 1:** Top 20 most significantly differentially expressed metabolites in galloflavin-treated boar sperm.

Metabolite	Log_2_(FC)	q-Value	VIP	Trend
Dihydrocaffeic acid	7.059216299	0.0000155619	2.265048631	up
Ascorbic acid	6.601847614	0.000001869	2.261476809	up
Thioguanine	5.088993588	0.005054894	2.194754951	up
Nicotinamide-N-oxide	4.865928826	0.000216815	2.207323893	up
Glycocholic acid	4.243191869	0.003951384	2.255444956	up
(R)-(-)-Mandelic acid	3.795786935	0.000182262	2.269121872	up
Caffeic acid	3.534042358	0.000234693	2.263952171	up
3-Hydroxyphenylacetic acid	3.515849734	0.001052035	2.260787843	up
Mandelic acid	3.515849734	0.001052035	2.260787843	up
P-Hydroxyphenyl acetic acid	3.515849734	0.001052035	2.260787843	up
Guanidineacetic acid	−1.233574996	0.000007168	2.208151633	down
Ala-Pro	−1.274703245	0.000014147	2.196194463	down
Barbituric acid	−1.533946617	0.000182262	2.227920112	down
Tyr-Tyr	−1.77389108	0.000452726	2.212834688	down
2-Pyrrolidineacetic acid	−2.142316674	0.000182262	2.110546771	down
Pantetheine	−2.146828496	0.000486023	2.002972538	down
Aconitic acid	−2.218104322	0.003195008	2.128753558	down
O-Acetyl-L-homoserine	−2.687479085	0.000606282	2.26538787	down
LPS (20:4/0:0)	−2.787999931	0.000383127	2.266873473	down
Imidazole-4-methanol	−3.65427166	0.000543418	2.256943795	down

## Data Availability

The original contributions presented in this study are included in the article/Supplementary Material. Further inquiries can be directed to the corresponding author.

## Supplementary Materials

The following supporting information can be downloaded at https://www.mdpi.com/article/10.3390/antiox15091132/s1, Figure S1: Western blot analysis of guanidinoacetate N-methyltransferase (GAMT) and mitochondrial creatine kinase (MtCK) protein expression in fresh boar sperm (Fs). β-tubulin was used as a loading control. Representative blots from three independent experiments are shown.
