# Supplementary material for: LDHC Hyperacetylation Disrupts the Guanidinoacetic Acid–Creatine Axis Underlying Cryoinjury in Boar Sperm: Rescue by GAA Supplementation
Source: Antioxidants (Basel). 2026 Sep 7;15(9):1132. doi: 10.3390/antiox15091132 (PMC13603231; doi:10.3390/antiox15091132)
Supplement: Supplementary file 1 [file antioxidants-15-01132-s001.zip › antioxidants-4530863-supplementary.pdf]

Supplemental Material

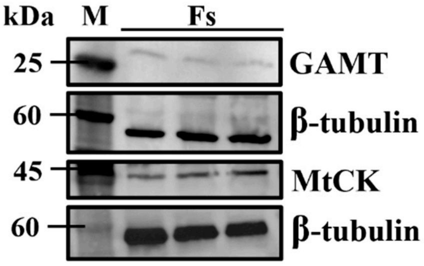

Figure S1. Western blot analysis of guanidinoacetate N-methyltransferase (GAMT) and mitochondrial creatine kinase (MtCK) protein expression in fresh boar sperm (Fs). β-tubulin was used as a loading control. Representative blots from three independent experiments are shown.
